# A systematic review: normative reference values of the median nerve cross-sectional area using ultrasonography in healthy individuals

**DOI:** 10.1038/s41598-022-13058-8

**Published:** 2022-06-02

**Authors:** Audrey Jing Ting Ng, Ramya Chandrasekaran, Ashutosh Prakash, Sreenivasulu Reddy Mogali

**Affiliations:** 1 Lee Kong Chian School of Medicine, Nanyang Technological University Singapore, 11 Mandalay Road, 308232 Singapore, Singapore; 2 Diagnostic Radiology, Tan Tock Seng Hospital, Singapore, Singapore

**Keywords:** Anatomy, Neurology

## Abstract

Median nerve cross-sectional area (CSA) was used for screening and diagnosis of neuropathy, but few studies have suggested reference range. Hence, this systematic review was performed to evaluate a normative values of median nerve CSA at various landmarks of upper limb based on ultrasonography. PubMed and Web of science were used to search relevant articles from 2000 to 2020. Forty-one eligible articles (2504 nerves) were included to access median nerve CSA at different landmarks (mid-arm, elbow, mid-forearm, carpal tunnel (CT) inlet and CT outlet). Data was also stratified based on age, sex, ethnicity, geographical location, and method of measurement. Random effects model was used to calculate pooled weighted mean (95% confidence interval (CI), [upper bound, lower bound]) at mid-arm, elbow, mid-forearm, CT inlet and outlet which found to be 8.81 mm^2^, CI [8.10, 9.52]; 8.57 mm^2^ [8.00, 9.14]; 7.07 mm^2^ [6.41, 7.73]; 8.74 mm^2^ [8.45, 9.03] and 9.02 mm^2^ [8.08, 9.95] respectively. Median nerve CSA varies with age, geographical location, and sex at all landmarks. A low (I^2^ < 25%) to considerable heterogeneity (I^2^ > 75%) was observed, indicating the variation among the included studies. These findings show that median nerve CSA is varying not only along its course but also in other sub-variables.

## Introduction

In the long course of the median nerve through the upper limb, there are multiple potential sites for lesions or injuries to occur. These lesions could be secondary to local aetiologies like trauma and compressive masses, or polyneuropathies such as diabetes mellitus, demyelinating diseases and rheumatoid arthritis causing nerve entrapment^1,2^. To evaluate the morphological changes of the median nerve in peripheral neuropathy, ultrasound technology has become a popular means^3^. Ultrasonography allowed nerve conduction studies to be supplemented via measurements of the nerve cross-sectional area (CSA).

Ultrasonography studies have commonly compared median nerve CSA between healthy controls and patients with peripheral neuropathy, thus establishing that CSA tend to increase in carpal tunnel syndrome and diabetic neuropathy^4,5^, whereas it is decreased in neurodegenerative conditions such as amyotrophic lateral sclerosis^6^. While these studies have stated the CSA cut-off for diagnosing their conditions, limited studies have suggested normative ranges of median nerve CSA which could potentially be applied for screening and diagnosis of other conditions. Therefore, this systematic review was aimed to report the normal median nerve CSA along its course at predetermined anatomical landmarks based on the reported ultrasound studies. This study also analysed the median nerve CSA in subgroup variables such as age, sex, ethnicity, geographical location, and method of CSA measurement. A common reference would be helpful for clinicians in improving screening and diagnostic methods for neuropathy of the median nerve.

## Materials and methods

### Study design, search strategy and selection process

This is a non-interventional systematic review of published articles from online databases to evaluate the range of median nerve CSA in the upper limb using ultrasonography. Article selection process was conducted and documented with the Preferred Reporting Items for Systematic reviews (PRISMA) and Meta-Analyses guidelines^7^. Using broader search terms, authors systematically searched articles from electronic databases—PubMed and Web of Science until 31 December 2020. The keywords used for the search strategy were “Median nerve”, “reference values” or “normative range”, “ultrasound imaging” and/or “ultrasonography”.

The studies were selected and included as per following eligibility criteria.

**Figure 1:**
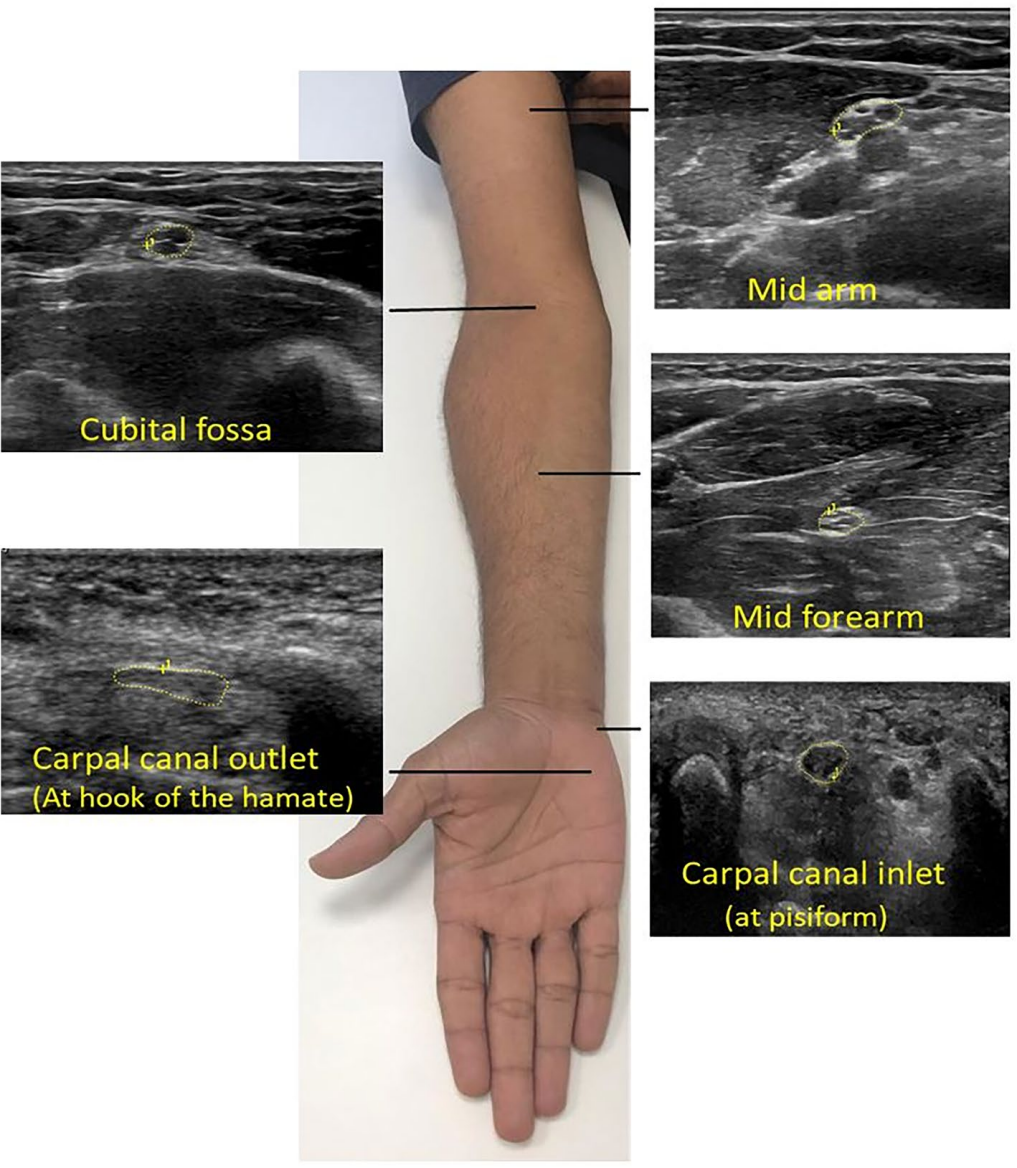
Ultrasound images and direct tracing of the median nerve cross sectional area at five different points of the right upper limb. A High-frequency 6–5 MHz linear array transducer was used (Logiq Eg, GE healthcare, WI, USA).

**Figure 2:**
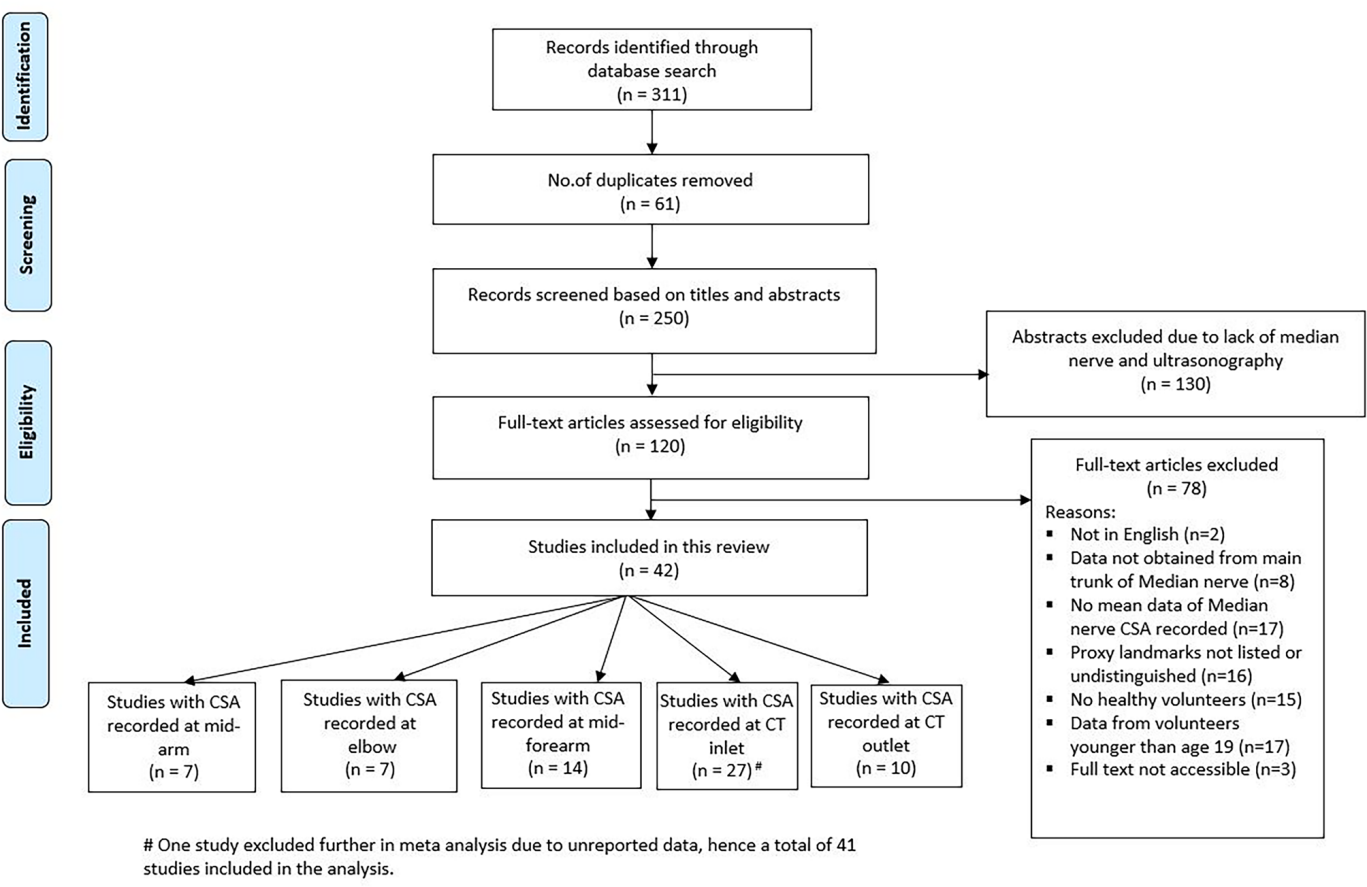
PRISMA Flowchart on steps for inclusion and exclusion of articles for review.

1. Peer-reviewed studies, written and published in the English language were included. Non-English articles, case reports, conference abstracts, editorial letters, expert opinion, articles without primary datas or incomplete results were excluded^8^. Given the development of ultrasound technology over the last 20 years^3^, empirical studies published from the year 2000 onwards were considered.
2. Articles were screened and included for the preliminary review that encompassed the primary variable of median nerve CSA, measured using ultrasonography in healthy volunteers, including control populations, at minimally one of the five proxy landmarks: the mid-arm, elbow (cubital fossa), mid-forearm, carpal tunnel (CT) inlet (level of the pisiform), and CT outlet (level of the hook of the hamate) (Fig. 1). These landmarks were the most common sites for measuring median nerve CSA.
3. Studies were excluded if the mean CSA was not reported, not obtained from main trunk of median nerve with the proxy landmarks not listed or undistinguished, and/or if data was obtained from pregnant women, nursing mothers or patients with history of neuropathy or associated risk factors. The list of risk factors associated with neuropathy is reported in the Supplementary Table 1.
4. Study subjects were assessed with minimal age of 19 years because epiphyseal closure in the longer bones of upper limb is expected to happen by this age^9^.
5. Duplicated records of articles were excluded, remaining records were screened by titles and abstracts according to the eligibility criteria described above. Subsequently, articles included from screening were assessed in full text for review and the reasons for exclusion were noted. The flowchart for study identification and evaluation is shown in the Fig. 2.

### Data extraction

Datas from the included articles were manually extracted and reviewed as per PRISMA guidelines. Sample size, mean, standard deviation and range of median nerve CSA for each of the anatomical landmark were recorded in Microsoft excel 2010 spreadsheet (Microsoft Corp., Redmond, WA) for the analysis. Secondary variables such as age, sex, geographical location, ethnicity, and method of measurement, were also noted for further stratification of the CSA. Some studies did not report data based on ethnicity, hence meta-analysis was reported as “unspecified ethnicity” and calculations were performed based on geographical location. The methods of measuring median nerve CSA and the boundary defining the median nerve was also noted, where this information was explicitly stated in the included studies. Information on positioning of upper limb, the number of operators for ultrasonic measurements was also recorded to supplement the discussion of results.

### Statistical analysis

Statistical analyses were performed using Open Meta software using R console (CEBM, Brown University)^10^. The data from the included articles were noted as continuous variables and the analysis was performed using mean, standard deviation and reported sample size (number of median nerves). The overall mean CSA was calculated for the articles that reported the mean value of CSA for subpopulations (for example male and female), using this equation—*Overall mean CSA* = *[sum of (N* × *mean CSA)] ÷ sum of N*, where N represents the number of upper limbs/median nerves measured^11^. Likewise, for the studies where the standard deviations were not provided, standard deviation was calculated manually using interquartile range, median and/or *P* values^12,13^.

The frequency of variations from the included studies were computed from weighted mean estimates using DerSimonian–Lard method^14,15^. A continuous random-effects model was used for the meta-analysis. The measures for all included studies were calculated at 95% confidence intervals (CI), showing the upper and lower boundary limits. Statistical heterogeneity of the pooled weighted mean was quantified using chi square $x$^2^ (represented by Cochran’s Q and *P* value) and I^2^ range, interpreted using Cochrane Handbook^16^. The I^2^ statistic measured the degree of inconsistency among the studies, indicated by the percentage of total variation effects. The thresholds for I^2^ were indicated as low or might not important (I^2^ < 25%), moderate (I^2^ = 30–60%), substantial (I^2^ = 50–90%) and a considerable heterogenicity (I^2^ = 75–100%)^17^. In addition, a Chi square test with Cochran’s Q, P < 0.10 was also considered for heterogenecity^17^.

In addition, appropriate subgroup analysis was performed to make comparisons and to identify the sources of heterogeneity. The variability was investigated based on sex, geographical location, ethnicity, and method of measurement with the datas stratified for each anatomical landmark in upper limb measuring median nerve CSA. Majority of the studies did not report median nerve CSA based on age groups, instead the overall mean age was available. Hence, for the purpose of analysis, we classified the age groups as young (19–40 years), middle age (41–65) and elderly (> 65) based on the mean age reported from the eligible studies. There were no clear scientific guidelines on the classification of age into different groups in the context of the evaluation of the CSA in human peripheral nerves such as median nerve. The age classification reported in this study is consistent in a clinicopathological study involving the gastric adenocarcinoma patients from different age groups^18^. The I^2^ statistic, weighted mean and standard error was calculated for each subgroup(s) at 95% confidence intervals based on Cochrane guidelines^17^.

### Quality and risk of bias assessment

The quality of the included studies was evaluated using Anatomical Quality Assessment (AQUA) tool^19^. This reporting is important as the poorly reported studies with lack of information may decrease the reliability, thus increase the potential risk of bias^19^. Studies were probed individually and judged by assessing five domains—objectives and characteristics of the study/subjects, design of the research study, methodology, description of anatomical structures and reporting of results. AQUA assessment guidelines define “low” as low risk of bias when the reported information is sufficient to reproduce. Studies that reported ambiguous and missing baseline information were considered to lack proper quality, hence they were ranked as “High” and “Unclear” risk of bias. Investigators #1 and #2 rated the included studies independently and all the disagreements were resolved through detailed discussion among the investigators.

### Ethical approval

All procedures followed were in accordance with the ethical standards of the responsible committee on human experimentation (institutional and national) and with the Helsinki Declaration of 1964 and later versions.

### Informed consent

Informed consent is not relevant in this case as this is a non-interventional literature review of published articles from online databases.

## Results

The initial literature search screened and potentially identified a total of 311 articles (158 records in PubMed and 153 in Web of Science), searched up to 31st December 2020. Subsequently, 61 duplicates were removed. The remaining 250 articles were scrutinized based on titles and abstracts. 130 papers were excluded further as their abstracts lacks the information on the median nerve and ultrasonography. From this, 120 potentially eligible articles were identified and 78 were excluded further (studies neither does not have full text nor failed to meet inclusion criteria), resulting in extraction of 42 studies meeting the inclusion criteria. Among the 42 studies, one study^20^ was excluded for the meta-analysis due to missing descriptive statistics information and hence 41 studies, reporting 2504 median nerves in 1614 healthy individuals were used for final systematic review. The number of articles that presented the mean median nerve CSA were seven^6,21–25,31^, seven^4,22,23,26–29^, fourteen^4,6,22,24–27,29–35^, twenty-seven^5,21–23,26,27,36,37,39–56,58^ and ten ^5,21,26,27,36,38,50,52,54,57^ based on mid-arm, elbow, mid-forearm, CT inlet and CT outlet, respectively. The study identification process and PRISMA flowchart outlining the systematic article review is provided in Fig. 2.

### Assessment of risk of bias of individual studies

**Figure 3:**
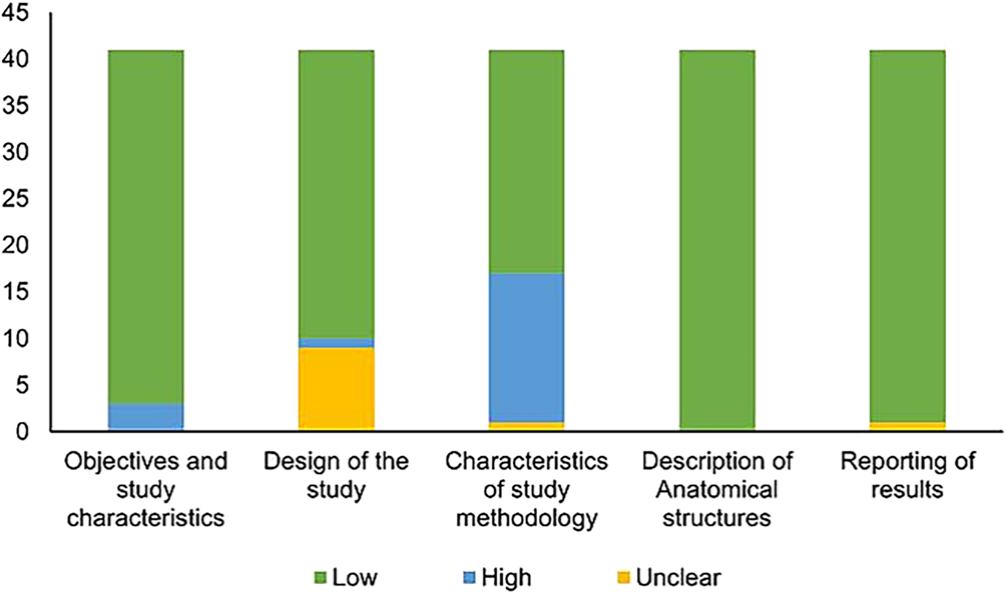
Quality assessment by Anatomical Quality Assessment tool (AQUA). Each study characteristics was accessed as “low,” “high,” or “unclear” risk of bias.

The risk was evaluated as “low” for majority of the included studies in all five domains. The summary of assessment of risk of bias is shown in Fig. 3 and detailed evaluation of individual studies is given in Supplementary Table 2. A few studies were rated as high risk of bias in domain one (objectives and study characteristics) and domain three (characteristics of study methodology).

### Median CSA by anatomical landmarks

The pooled weighted mean and 95% CI [upper bound, lower bound] of the median nerve CSA at the mid-arm, elbow, mid-forearm, CT inlet and outlets were 8.81 mm^2^, CI [8.10, 9.52]; 8.57 mm^2^, CI [8.00, 9.14]; 7.07 mm^2^, CI [6.41, 7.73]; 8.74 mm^2^, CI [8.45, 9.03] and 9.02 mm^2^, CI [8.08, 9.95], respectively. This indicates that the median nerve CSA is variable at different points in its course, such that it decreases from the mid-arm to mid-forearm and then increases distally at the CT inlet and outlet. A significant Cochran Q statistic I^2^ > 90% for each landmark showed the presence of considerable heterogeneity variation among the included studies (Table 1). A summary of weights of each individual study, categorised based on respective anatomical landmarks is given in Supplementary Table 3.

**Table 1:** Summary of pooled weighted mean of median nerve CSA reported based on anatomical landmarks of the upper limb. Total number of included studies were 41. CSA = cross sectional area. CI = confidence intervals. *Heterogeneity (I^2^) *P* values of Cochran Q statistic is *P* < 0.001 in all landmarks, except Mid-arm with *P* < 0.01.

Anatomical landmark	Number of studies included	Study details	Number of Median nerves evaluated	CI at 95%*				
				Lower bound	Upper bound	Weighted mean CSA (mm^2^)	I^2^ (%)	Standard error
Mid-arm	7	^6,21–25,31^	631	8.10	9.52	8.81	96.78	0.36
Elbow	7	^4,22–23,26–29^	625	8.00	9.14	8.57	94.47	0.29
Mid-forearm	14	^4,6,22,24–27,29–35^	844	6.41	7.73	7.07	98.67	0.34
CT inlet	27	^5,21–23,26–27,36–37,39–56,58^	1863	8.45	9.03	8.74	97.43	0.15
CT outlet	10	^5,21,26–27,36,38,50,52, 54,57^	634	8.08	9.95	9.02	98.36	0.48

### Median nerve CSA by age

A total of thirty-four studies reported the overall mean age of the participants which ranged from 21 to 82. The median nerve CSA for age groups at different landmarks were summarised in Table 2. At mid-arm and CT outlet, middle age group CSA appeared to be higher compared to other groups. In younger adults, CSA at mid-forearm was higher compared to other groups. Interestingly, the CSA at elbow and CT inlet were similar in young and middle age groups. A considerable heterogeneity (I^2^ > 90% with *P* value < 0.001) was observed in most of the studies.

**Table 2:** Summary of pooled weighted mean of median CSA (n = 34 studies) based on mean age groups at various anatomical landmarks of the upper limb. CSA = cross sectional area. NA indicates the inclusion of only one study for the analysis, hence heterogenicity (I^2^) cannot be calculated. ^#^Indicates inclusion of study from same authors/research group.

Anatomical landmark			Number of studies included	Study details		Mean age range of included studies	Number of median nerves evaluated		CI at 95%			Weighted mean CSA (mm^2^)	I^2^ (%)	Standard error
									Lower bound		Upper bound			
Mid-arm			1	^23^		19–40	120		7.89		8.50	8.20	NA	0.15
			3	^6,22,25^		41–65	209		9.20		9.55	9.37	0.00^#^	0.09
			1	^24^		> 65	14		7.81		9.91	8.86	NA	0.53
Elbow			1	^23^		19–40	120		8.71		9.49	9.10	NA	0.20
			4	^22,27–29^		41–65	380		8.10		9.67	8.89	92.71	0.40
			0	–		> 65	–		–		–	–	–	–
Mid-forearm			3	^31,33,35^		19–40	101		6.85		12.22	9.54	98.97	1.36
			6	^6,22,25,27,29,30^		41–65	387		6.43		7.62	7.03	94.13	0.30
			3	^24,34,35^		> 65	51		5.51		10.24	7.87	95.04	1.21
CT inlet			4	^23,50,53–54^		19–40	270		7.85		9.73	8.79	93.84	0.48
			18	^5,22,27,36–37,39–43,45–49,52,55–56^		41–65	1211		8.48		9.31	8.89	97.18	0.21
			0	–		> 65	–		–		–	–	–	–
CT outlet			1	^54^		19–40	40		7.50		8.49	8.00	NA	0.25
			5	^5,27,36,38,52^		41–65	197		8.94		10.62	9.78	90.47	0.42
			0	–		> 65	–		–		–	–	–	–

### Median CSA by sex

Seven studies^23,29,32,37,48,50,52^ involving 534 and 614 median nerves were reported for males and females based on different landmarks (Table 3). The weighted mean median nerve CSA of males showed generally larger than females at all the landmarks (> 1 mm^2^) except for CT inlet and mid-forearm where CSA values were similar (< 0.5 mm^2^).

**Table 3:** Subgroup analysis (of included studies, n = 7) based on sex at different anatomical landmarks measuring median nerve CSA. CSA = cross sectional area. CI = confidence intervals. NA indicates the inclusion of only one study for the analysis, hence heterogenicity (I^2^) cannot be calculated.

Anatomical landmark	Number of studies included	Study details	Sex	Number of Median nerves evaluated	CI at 95%				
					Lower bound	Upper bound	Weighted mean CSA (mm^2^)	I^2^ (%)	Standard error
Mid-arm	1	^23^	Males	58	8.56	9.44	9.00	NA	0.22
	1	^23^	Females	62	7.05	7.75	7.40	NA	0.21
Elbow	2	^23,29^	Males	113	9.14	10.51	9.83	74.98	0.35
	2	^23,29^	Females	107	7.56	9.32	8.44	86.32	0.45
Mid-forearm	2	^29,32^	Males	155	4.04	8.35	6.20	99.16	1.10
	2	^29,32^	Females	145	3.25	8.34	5.79	99.37	1.30
CT inlet	3	^23,37,50^	Males	178	8.38	9.66	9.02	77.80	0.33
	5	^23,37,48,50,52^	Females	230	8.14	9.88	9.01	88.97	0.44
CT outlet	1	^50^	Males	30	8.77	11.21	9.99	NA	0.31
	2	^50,52^	Females	70	7.98	9.05	8.51	0.00	0.27

### Median nerve CSA by geographical location and ethnicity

Forty-one studies reported median nerve CSA from different geographical locations. Majority of the studies (n = 29) did not report the sub-population ethnicity and hence the meta-analysis was performed based on geographical location reported in the studies (Table 4). At mid-arm, the mean median nerve CSA was smaller for Europe (7.00 mm^2^) compared to Oceania (9.28 mm^2^) and Asia (9.06 mm^2^) studies. At elbow, the mean median nerve CSA was found higher for Europe (9.20 mm^2^) compared to Asia (8.27 mm^2^). At mid-forearm, the mean median nerve CSA appears to be larger in Americas (9.38 mm^2^) followed by Europe (7.15 mm^2^), Asia (6.24 mm^2^) and Oceania (5.91 mm^2^). Interestingly, the mean median nerve CSA for CT inlet seems to be similar for middle east (8.77 mm^2^), Oceania (8.71 mm^2^), Europe (8.90 mm^2^) and Asia (8.68 mm^2^) studies. However, the mean median nerve CSA at CT outlet is larger for Asia (9.20 mm^2^) compared to the middle East (8.18 mm^2^) reports. These results indicate that there is a considerable variation of median nerve CSA among the different geographical locations for different landmarks of the upper limb except CT inlet (< 0.25mm^2^).

**Table 4:** Subgroup analysis (of included studies, n = 41) of median nerve CSA based on geographical location and ethnicity (of included studies, n = 12) at different anatomical landmarks. CSA = cross sectional area. CI = confidence intervals. NA indicates the inclusion of only one study for the analysis, hence heterogenicity (I^2^) cannot be calculated. Unspecified indicates study that has not mentioned sub-population group and hence meta-analysis was performed based on geographical location.*Indicates Asia excluding Middle Eastern countries.

Anatomical landmark	Number of studies included	Study details	Geographical location	Ethnicity	Number of Median nerves evaluated	CI at 95%				
						Lower bound	Upper bound	Weighted mean CSA (mm^2^)	I^2^ (%)	Standard error
Mid-arm	3	^6,24,25^	Oceania	Unspecified	29	8.58	9.98	9.28	0.00	0.36
	1	^31^	Europe	Unspecified	48	6.60	7.40	7.00	NA	0.20
	3	^21–23^	Asia*	**Japanese, Chinese and Unspecified**	554	8.41	9.71	9.06	96.8	0.33
	1	^23^		Japanese only	120	7.90	8.50	8.20	NA	0.16
	1	^21^		Chinese only	240	9.42	9.72	9.57	NA	0.08
Elbow	2	^28,29^	Europe	Unspecified	150	8.94	9.46	9.20	NA	0.13
	5	^4,22–23,26–27^	Asia*	**Japanese, Chinese, Indians and Unspecified**	475	7.58	8.95	8.27	94.49	0.35
	1	^23^		Japanese only	120	8.71	9.45	9.10	NA	0.01
	1	^26^		Chinese only	80	8.43	8.87	8.65	NA	0.03
	1	^4^		Indians only	45	6.48	7.32	6.90	NA	0.21
Mid-forearm	3	^29–31^	Europe	Unspecified	193	6.84	7.45	7.15	68.39	0.16
	3	^6,24–25^	Oceania	Unspecified	29	5.43	6.38	5.91	0.00	0.24
	5	^4,22,26,27,32^	Asia*	**Chinese, Indians and Unspecified**	555	5.27	7.22	6.24	99.22	0.50
	1	^26^		Chinese only	80	5.50	5.90	5.70	NA	0.01
	2	^4,32^		Indians only	245	3.97	7.31	5.64	98.54	0.85
	3	^33–35^	Americas	**Caucasian and Unspecified**	67	7.34	11.43	9.38	95.36	1.05
	1	^34^		Caucasian only	17	7.30	9.10	8.20	NA	0.03
CT inlet	7	^44–45,48–49,51,52,54^	Middle East	Unspecified	248	8.11	9.43	8.77	92.78	0.34
	2	^43,58^	Oceania	Unspecified	63	7.58	9.84	8.71	97.15	0.58
	6	^37,41–42,47,53,56^	Europe	**Caucasian and Unspecified**	503	8.46	9.34	8.90	86.39	0.23
	1	^41^		Caucasian only	56	8.03	8.97	8.50	NA	0.24
	12	^5,21–23,26–27,36,39–40,46,50,55^	Asia*	**Chinese, Japanese and Unspecified**	1049	7.96	9.4	8.68	98.56	0.37
	6	^21,26,36,39,46,55^		Chinese only	568	7.09	9.14	8.12	98.79	0.52
	1	^23^		Japanese only	120	8.20	8.80	8.50	NA	0.16
CT outlet	2	^52,54^	Middle East	Unspecified	80	7.63	8.73	8.18	24.43	0.28
	8	^5,21,26–27,36,38,50,57^	Asia*	**Chinese and Unspecified**	554	8.10	10.29	9.20	98.72	0.56
	4	^21,26,36,38^		Chinese only	400	7.30	10.65	8.97	99.30	0.85

Regarding ethnicity, only twelve^4,21,23,26,32,34,36,38,39,41,46,55^ articles clearly reported the ethnicity of the sub-populations. Of this, seven articles reported for Chinese^21,26,36,38,39,46,55^, two for Caucasian^34,41^, two for Indians^4,32^ and one article presented for Japanese^23^. At mid-arm, the pooled weighted mean CSA of Chinese (9.57 mm^2^) is more than the Japanese (8.20 mm^2^). The mean CSA of median nerve appears to be smallest at elbow in Indian population (6.90 mm^2^), compared to the Japanese (9.10 mm^2^) and Chinese (8.65 mm^2^). Likewise at mid-forearm, the mean CSA of median nerve is higher in Caucasian (8.20 mm^2^) compared to the Indians (5.64 mm^2^) and Chinese (5.70 mm^2^) subjects. At CT inlet, the Caucasian (8.50 mm^2^), Japanese (8.50 mm^2^) and Chinese (8.12 mm^2^) CSA values were similar. The median nerve mean CSA at CT outlet (8.97 mm^2^) was available for Chinese subjects only. These findings represent the differences in median nerve CSA along its course of upper limb for different ethnicities (Table 4).

### Median nerve CSA based on method of measurement

Thirty-one articles reported measurement of CSA by the method of direct tracing method, out of which 26 articles^5,6,21–25,27–30,32,36–47^ traced CSA without the hyperechogenic rim of the epineurium of the median nerve. The echogenic rim of the epineurium is reported but did not provide any image of ultrasound trace for interpretation^34^. Seven articles measured CSA using the elliptical selection (CSA = diameter × width × π), out of which one article^53^ included the epineurium. Two articles^49,53^ inferred to have excluded the epineurium, while studies from Bayrak et al., (2007)^54^ and Lu et al., (2015)^55^ did not provide any ultrasound images of median nerve CSA for inference^54,55^. Two studies^35,36^ used unique methods of measurement (freehand lasso tool on the Adobe Photoshop and automatically using integrated software) but failed to mention if the epineurium was included or not. One article^31^ reported the measurement of direct tracing within the hyperechoic epineurial rim and continuous trace tool within the outer epineurial rim. In summary, direct tracing was most reported method that used to measure the CSA and this method yielded higher CSA value at CT outlet (9.24 mm^2^), followed by mid-arm (9.14mm^2^), CT inlet (8.96 mm^2^), elbow (8.57 mm^2^) and mid-forearm (6.73 mm^2^) compared to the other methods. Likewise, other methods such as Freehand lasso adobe photoshop produced abnormally higher CSA value in mid-forearm (11.75 mm^2^). In conclusion, considerable heterogeneity (I^2^ > 90%) was observed for this variable at all landmarks, except at CT outlet reported a moderate heterogeneity (I^2^ = 43.12%) when measured using elliptical selection method (Table 5).

**Table 5:** Subgroup analysis of included studies (n = 41), stratified based on method of measuring CSA at different anatomical landmarks measuring median nerve. CSA = cross sectional area. CI = confidence intervals.NA indicates the inclusion of only one study for the analysis, hence heterogenicity (I^2^) cannot be calculated.

Anatomical landmark	Number of studies included	Study details	Method of measurement	Number of Median nerves evaluated	CI at 95%				
					Lower bound	Upper bound	Weighted mean CSA (mm^2^)	I^2^ (%)	Standard error
Mid-arm	6	^6,21,22–25^	Direct tracing	583	8.62	9.66	9.14	92.14	0.27
	1	^31^	Elliptical method, supplemented by direct tracing	48	6.60	7.40	7.00	NA	0.20
Elbow	7	^4,22–23,26–29^	Direct tracing	625	8.00	9.14	8.57	94.47	0.29
Mid-forearm	12	^4,6,22,24–27,29–30,32–34^	Direct tracing	773	6.03	7.42	6.73	98.60	0.35
	1	^31^	Elliptical selection, supplemented by direct tracing	51	6.68	7.12	6.90	NA	0.11
	1	^35^	Freehand lasso adobe photoshop	20	10.80	12.71	11.75	NA	0.49
CT inlet	21	^5,21–23,26–27,36–37,39–51^	Direct tracing	1612	8.58	9.34	8.96	97.49	0.19
	6	^49,52–55,58^	Elliptical selection	237	7.52	8.29	7.91	96.87	0.20
	1	^56^	Automatically using integrated software	55	8.34	8.86	8.60	NA	0.14
CT outlet	7	^5,21,26–27,36,38,50^	Direct tracing	537	8.05	10.43	9.24	98.90	0.61
	3	^52,54,57^	Elliptical selection	97	7.83	8.95	8.39	43.12	0.29

## Discussion

The current study systematically reviewed the existing literature to demonstrate the normative median nerve CSA along its course in the upper limb at various anatomical landmarks. The median nerve is one of the most clinically important and commonly afflicted nerves in the upper limb. Meta-analysis findings from this study generated weighted pooled results of median nerve CSA at different points as well as based on sub-group analysis. This evidence-based observation of normative median nerve CSA would be helpful to the clinicians, neuroradiologists, ultra-sonographers in clinical screening and diagnosis of the normal and pathological median nerve based on the ultrasonography studies.

### Analysis of CSA by landmark

Interestingly, the median nerve CSA was much higher at the mid- forearm^35^, CT inlet^48^ and CT outlet^38^ compared to the other studies for the same landmarks. The discrepancy in mean CSA at the mid-forearm is likely related to the deviation of methodology in measuring CSA^35^. While all other studies have used the ultrasound machine to either trace the echogenic boundary of the median nerve or input values for the ellipsoid formula, this study^35^ utilised computer software Adobe Photoshop to trace the median nerve. However, the detailed information on how the median nerve boundary defined was not available. On the other hand, the median nerve CSA at the CT inlet reported by Kaymak et al.^48^ can be further investigated, as the study reported the widest range of CSA (5.90–20.7 mm^2^) among the rest of the studies included in this review. The authors included the epineurium in its CSA measurement but were unable to elucidate the contributing factors behind this variation. Lastly, the study from Tsai et al.^38^ did not report the results of the median nerve conduction in the control population, thus investigators were unable to determine whether the control population included individuals with subclinical median nerve neuropathy.

Despite these anomalies, the overall measurement of median nerve CSA was reported to decrease in size, followed by an increase distally down the upper limb. The decrease of median nerve CSA in the proximal upper limb could be explained by the branching of the median nerve at the elbow as it provides an innervation to the forearm muscles, such as the pronator teres, flexor carpi radialis, flexor digitorum sublimis and palmaris longus^59^. The weighted pool estimates of median nerve CSA tends to be the smallest at the mid-forearm (7.07 mm^2^). This can be explained by the branching of the anterior interosseous nerve at the forearm which would further reduce the median nerve CSA in the mid-forearm before increasing again prior to reaching the carpal tunnel, as evidenced in cadaveric studies^60,61^. The distal increase of CSA could be possibly explained by the redistribution of nerve fibres into more fascicular bundles^62^. However, a cadaveric study by Perumal and Stringer^63^ showed no significant difference in the number of fascicles in the median nerve around the carpal tunnel area and the redistribution of fascicles affected the antero-posterior and transverse diameters, but not due to the CSA. Yet, the authors of the same study acknowledged the limitations such as the smaller sample size, age, and gender distribution of the samples^63^.

### Analysis of CSA by age

There is no uniform age classification to investigate the median nerve CSA changes in the normal subjects. This is a limitation to understand the progress of the median nerve CSA from young and elderly. However, we classified the age and analysed the changes in CSA based on the overall mean age reported in the studies. Except for elbow and CT inlet, the CSA was found to be varied for different age groups at different landmarks. This observation may be due to limited number of studies with sample size (Table 2) and different methods of CSA measurements. For example, at mid-forearm, Li et al. (2015) reported higher CSA in young age group 13.6 mm^2^ which is considerably higher compared to other studies for the similar age group. This could be due to use of adobe photoshop to trace the median nerve boundary instead of tracing method in the ultrasound machine. Another reason for varied CSA for different age groups at different landmarks might be due to accumulation of inter-fascicular adipose, connective tissue, redistribution of fascicles and compartmentalisation in the median especially at CT region^64,65^. One article^34^ reported mean age based on body mass index, highlighting that the weight considered as major parameter for predicting median nerve CSA. Chen et al. (2011)^21^ compared median nerve CSA at the mid-arm, CT inlet and CT outlet among different age groups and reported no statistical difference between the middle-aged and elderly groups. Hence, considering the limited number of articles that reported age range, further research is recommended to support both the trend in CSA at these landmarks and their causative mechanisms.

### Analysis of CSA by geographical location and ethnicity

Sub-group analysis based on geographical location showed a considerable variation of median nerve CSA among the different geographical locations for different landmarks of the upper limb except CT inlet. This indicates the CSA values reported in one part of the world may not be applicable to other geographical locations. So, it may be worth to have their own data of median CSA representing the country specific/ ethnicity. This sub-group analysis also suffers from the small number of available studies that explicitly reported median nerve CSA based on the geographical location and ethnicity. Therefore, future studies would be needed for determining the relationships of median nerve CSA across different ethnic groups.

### Analysis of CSA by sex

Comparison of median nerve CSA by sex indicated that males generally have larger CSA than females. A proposed reason could be males usually having greater weight and body mass index (BMI)^66^, which contribute to correlate with peripheral nerve CSA^67^. However, with limited number of eight articles reporting median nerve CSA by sex, more research has to be conducted in future to substantiate the finding from this review. Notably, Kaymak et al.^48^ presented a larger mean CSA of 11.5 mm^2^ at the CT inlet in females, which is larger than the mean CSA noted in both the male^23,37,50^ and female populations^23,37,50,52^. The reason for Kaymak et al. study^48^ deviating from the trend discussed above is uncertain, although it could be related to the reduced reliability of this study as discussed above. If data from this study was omitted, the mean CSA at the CT inlet in females would range from 7.94 to 8.83 mm^2^ as reported in these three articles^37,50,53^.

### Differences in method of measuring CSA and definition of CSA boundary

There was lack of standardised protocol in measuring median nerve CSA across all articles. This included the various upper limb positions that were adopted for the measurement in healthy volunteers, involving wrist hyperextension reported by Kaymak et al.^48^, and Li et al.^35^ who performed CSA measurements using computer Freehand lasso adobe photoshop software instead using ultrasound machine. Although there was variation in the method of measuring median nerve CSA, both direct tracing and ellipsoid formula (*CSA* = *diameter* × *width* × *π*) measurements yielded similar results^68^ and thus should not affect reliability of the findings in this review. On the other hand, although there was disparity in whether the epineurium was included within the median nerve CSA measurement, the significance of epineurium thickness in the comparison of median nerve CSA has hardly been discussed in current literature^69^. Furthermore, with ultrasonography being operator-dependant, only six articles^27,30,35,38,42,52^ reported using more than one operator and seventeen^5,6,20,24,25,30,39,40,42,43,45,46,49,50,52,53,57^ articles blinded their operators to reduce bias in interpreting measurements.

### Limitations of this review

Overall, this review analysed the mean median nerve CSA at different anatomical landmarks in the upper limbs of healthy individuals. However, it was limited by the presence of considerable heterogenicity among the included studies. This could be due to the slightly different anatomical variations among the healthy individuals and calculated across different geographical locations. Despite this observation, the source of heterogeneity was not identified even when subgroup analysis was performed based on age, sex, geographical location, and method of measurement. Although a very high heterogeneity was observed in most subgroups with an I^2^ range greater than 80%, we also found low to moderate variations (I^2^ = 0–50%). Results from the latter could be due to the inclusion of the studies from the same research group and limited number of studies.

Furthermore, the ranges of mean median nerve CSA calculated in this study were not categorized based on right and left hands, as other studies reported no significant bilateral differences regardless of hand dominance^48,70^. The median nerve CSA was also not stratified by BMI as this review aimed to report the range of normative median nerve CSA values applicable to asymptomatic individuals regardless of BMI. Authors believed that this information may be useful in establishing a standardised reference range for the screening and diagnosis of median nerve neuropathy or determining the threshold for treatment like surgical repairs of median nerve injuries. As different studies used different scanning protocols and measurement methods (direct tracing, freehand adobe photoshop and elliptical tracing) to obtain the ultrasound images, this may create bias in interpreting the measurements. Future studies may include establishing standardised protocols and methods to ensure data accuracy and repeatability when measuring median nerve CSA in various upper limb positions.

In addition to a study with limited sample size as low as seven^6^, there were paucity of articles reporting the detailed ethnicities and age range for subgroup analysis, which may over- or understate the CSA range.

Lastly, a publication bias might have occurred as authors restricted the literature search to English language. This might have limited the complete inclusion of all existing ultrasonography studies.

### Future research

Investigations could explore whether differences in median nerve CSA with or without epineurium of the median nerve can be considered, although this difference may be statistically relevant and important. Beyond the range of mean CSA, inclusion of other methods for reviewing normal median nerve CSA cut-offs between articles could potentially explored in future research such as comparing the ratio between wrist-to-forearm median nerve CSA or wrist circumference to median nerve CSA. Moreover, given that the CSA of the median nerve that comprised of area formed by fascicular bundles and non-fascicular area, ultrasound measurements and comparison of the ratio between these areas can be studied further in order to potentially establish the causal mechanisms behind the trends noted in this review. There are no clear scientific suggestions on how to categorize age groups when measuring CSA in human peripheral nerves such as the median nerve. This work might encourage future research on determining the median nerve CSA in the age categories suggested.

## Conclusion

This study reported the pooled weighted mean of median nerve CSA along its length (in normal subjects) as well as across geographical locations, age, sex, and method of measurements based on the ultrasonography studies. Our findings on normative CSA values may be considered as a resource for clinicians and surgeons as a cut-off point for investigating preclinical screening and diagnosing the median nerve neuropathy. However, further studies are recommended to obtain normative median nerve CSA values based on BMI across the various ethnic populations and different age groups using standardised protocols.

## Supplementary Information

Supplementary Information 1.

Supplementary Information 2.

Supplementary Information 3.

## Data Availability

The datasets used in this systematic study were taken from the published articles extracted from online databases such as PubMed and Web of Science.
