# Supplementary material for: A systematic review: normative reference values of the median nerve cross-sectional area using ultrasonography in healthy individuals
Source: Sci Rep. 2022 Jun 2;12:9217. doi: 10.1038/s41598-022-13058-8 (PMC9163181; doi:10.1038/s41598-022-13058-8)
Supplement: Supplementary file 1 — Supplementary Information 1. [file 41598_2022_13058_MOESM1_ESM.docx]

| Local Risk factors | Systematic risk factors |
| --- | --- |
| - Carpal tunnel syndrome in either upper limb - Trauma to ipsilateral upper limb e.g. fractures, dislocation - Surgery to ipsilateral upper limb - Compressive masses ipsilateral upper limb e.g. tumour, ganglion - Osteoarthritis in either upper limb | - Metabolic disorders e.g. diabetes mellitus, alcoholism, hyper/hypothyroidism - Autoimmune and inflammatory disorders e.g. chronic inflammatory demyelinating polyneuropathy, Guillain Barré Syndrome, rheumatoid arthritis, systemic lupus erythematosus, vasculitis - Demyelination disorders e.g. chronic inflammatory demyelinating polyneuropathy (CIDP) - Infection affecting nerves e.g. leprosy |

**Supplementary Table 1**: Risk factors associated with neuropathy
