# Supplementary material for: A systematic review: normative reference values of the median nerve cross-sectional area using ultrasonography in healthy individuals
Source: Sci Rep. 2022 Jun 2;12:9217. doi: 10.1038/s41598-022-13058-8 (PMC9163181; doi:10.1038/s41598-022-13058-8)
Supplement: Supplementary file 2 — Supplementary Information 2. [file 41598_2022_13058_MOESM2_ESM.docx]

**Supplementary table 2**: Quality assessment by Anatomical Quality Assessment tool (AQUA).

Each study characteristics was accessed as “low,” “high,” or “unclear” risk of bias in five domains - Objectives and characteristics of the study/subjects, design of the research/study, characteristics of study methodology, description of anatomical structures and reporting of results.

Studies were rated as high or unclear if they did not

^a, b^ explicitly define the inclusion and exclusion criteria for the selection of subjects

^c, d^ mention the number of ultrasonographic examiners and/or whether they are completely/partially blinded with the patient information

^e^ provide details on epineurium boundary measuring median nerve CSA and/or lack of measures to establish intra/interobserver reliability

^f^ clearly explained in the results summary as why only some subjects were excluded

| **Study details** | **Country of study** | **Total number of Median nerves evaluated** | **Number of healthy controls** | **Risk of Bias (AQUA)** | | | | |
| --- | --- | --- | --- | --- | --- | --- | --- | --- |
|  |  |  |  | **Objectives and Study characteristics** | **Design of the study** | **Characteristics of study methodology** | **Description of Anatomical structures** | **Reporting of results** |
| Singh et al., 2019^4^ | India | 45 | 45 | Low | Unclear^c^ | High^e^ | Low | Low |
| Kwon et al., 2008^5^ | Korea | 41 | 29 | Low | Low | Low | Low | Low |
| Pelosi et al.,2017^6^ | New Zealand | 7 | 7 | High^a^ | Low | Low | Low | Low |
| Chen et al., 2011^21^ | China | 240 | 120 | Low | Low | Low | Low | Low |
| Won et al., 2013^22^ | Korea | 194 | 97 | Low | Low | Low | Low | Low |
| Sugimoto et al., 2013^23^ | Japan | 120 | 60 | Low | Low | Low | Low | Low |
| Pelosi et al., 2018^24^ | New Zealand | 14 | 14 | High^a^ | High^d^ | High^e^ | Low | Low |
| Mulroy et al., 2018^25^ | New Zealand | 8 | 8 | Low | Low | High^e^ | Low | Low |
| Niu et al., 2020^26^ | China | 80 | 80 | Low | Unclear^c^ | High^e^ | Low | Low |
| Jang et al., 2014^27^ | Korea | 36 | 18 | Low | Low | Low | Low | Low |
| Grimm et al., 2017^28^ | Germany | 50 | 50 | Low | Low | Low | Low | Low |
| Grimm et al., 2018^29^ | Germany | 100 | 100 | Low | Low | Low | Low | Low |
| Grimm et al.,2014^30^ | Germany | 42 | 21 | High^b^ | Low | Low | Low | Low |
| Rosmalen et al., 2019^31^ | Netherlands | 51 | 51 | Low | Unclear^c^ | High^e^ | Low | Unclear^f^ |
| Bathala et al., 2014^32^ | India | 200 | 100 | Low | Low | High^e^ | Low | Low |
| Lothet at al., 2019^33^ | USA | 30 | 15 | Low | Unclear^c^ | High^e^ | Low | Low |
| Cartwright et al., 2013^34^ | USA | 17 | 20 | Low | Low | Low | Low | Low |
| Li et al., 2015^35^ | Americas | 20 | 10 | Low | Low | Unclear^e^ | Low | Low |
| Wang et al., 2008^36^ | Taiwan | 40 | 20 | Low | Low | Low | Low | Low |
| Kerasnoudis et al., 2013^37^ | Germany | 150 | 75 | Low | Low | Low | Low | Low |
| Tsai et al., 2013^38^ | Taiwan | 40 | 20 | Low | Low | Low | Low | Low |
| Horng et al., 2014^39^ | Taiwan | 106 | 53 | Low | Low | High^e^ | Low | Low |
| Kim et al., 2014^40^ | Korea | 30 | 19 | Low | Low | High^e^ | Low | Low |
| Böhm et al., 2014^41^ | Europe | 56 | 56 | Low | Low | Low | Low | Low |
| Kerasnoudis et al., 2015^42^ | Germany | 150 | 75 | Low | Low | Low | Low | Low |
| Borire et al., 2016^43^ | Australia | 40 | 20 | Low | Unclear^c^ | Low | Low | Low |
| Arslan et al., 2018^44^ | Turkey | 21 | 11 | Low | Low | Low | Low | Low |
| Atan et al., 2018^45^ | Turkey | 50 | 50 | Low | Low | Low | Low | Low |
| Chang et al., 2009^46^ | China | 59 | 32 | Low | Low | Low | Low | Low |
| Kleermaeker et al., 2019^47^ | Netherlands | 42 | 42 | Low | Low | Low | Low | Low |
| Kaymak et al., 2008^48^ | Turkey | 38 | 19 | Low | Low | High^e^ | Low | Low |
| Rahmani et al., 2011^49^ | Iran | 41 | 41 | Low | Low | High^e^ | Low | Low |
| Kim et al., 2012^50^ | Korea | 60 | 60 | Low | Low | Low | Low | Low |
| Cingoz et al., 2018^51^ | Turkey | 18 | 9 | Low | Low | High^e^ | Low | Low |
| Keles et al., 2005^52^ | Turkey | 40 | 20 | Low | Low | High^e^ | Low | Low |
| Ažman et al., 2009^53^ | Croatia | 50 | 25 | Low | Unclear^c^ | High^e^ | Low | Low |
| Bayrak et al., 2007^54^ | Turkey | 40 | 20 | Low | Low | Low | Low | Low |
| Lu et al., 2015^55^ | China | 43 | 40 | Low | Unclear^b,c^ | Low | Low | Low |
| Kele et al., 2003^56^ | Germany | 55 | 33 | Low | Low | High^e^ | Low | Low |
| Kuo et al., 2001^57^ | Taiwan | 17 | 17 | Low | Unclear^c^ | High^e^ | Low | Low |
| Wilkinson et al., 2001^58^ | Australia | 23 | 12 | Low | Unclear^c^ | Low | Low | Low |
