# Supplementary material for: A systematic review: normative reference values of the median nerve cross-sectional area using ultrasonography in healthy individuals
Source: Sci Rep. 2022 Jun 2;12:9217. doi: 10.1038/s41598-022-13058-8 (PMC9163181; doi:10.1038/s41598-022-13058-8)
Supplement: Supplementary file 3 — Supplementary Information 3. [file 41598_2022_13058_MOESM3_ESM.docx]

**Supplementary Table 3:**

Summary of weights of each study, stratified based respective anatomical landmarks – Mid-arm, Elbow, Mid-forearm, CT inlet and CT outlet.

The weights are calculated using continuous random effects model at 95% CI.

| Anatomical landmark | Study details | Total number of Median nerves evaluated | Statistics for each study | | |
| --- | --- | --- | --- | --- | --- |
|  |  |  | Lower limit | Upper limit | Weighted mean (%) |
| Mid-arm | [6] | 7 | 7.94 | 11.00 | 9.50 |
|  | [21] | 240 | 9.42 | 9.72 | 16.90 |
|  | [22] | 194 | 9.19 | 9.55 | 16.85 |
|  | [23] | 120 | 7.90 | 8.50 | 16.52 |
|  | [24] | 14 | 7.81 | 9.91 | 12.44 |
|  | [25] | 8 | 8.52 | 10.88 | 11.61 |
|  | [31] | 48 | 6.60 | 7.40 | 16.18 |
| Elbow | [4] | 45 | 6.48 | 7.32 | 15.87 |
|  | [22] | 194 | 7.93 | 8.33 | 16.98 |
|  | [23] | 120 | 8.71 | 9.49 | 16.03 |
|  | [26] | 80 | 8.43 | 8.87 | 16.93 |
|  | [27] | 36 | 6.55 | 14.26 | 1.93 |
|  | [28] | 50 | 8.78 | 9.62 | 15.88 |
|  | [29] | 100 | 8.87 | 9.53 | 16.38 |
| Mid forearm | [4] | 45 | 6.12 | 6.88 | 7.47 |
|  | [6] | 7 | 4.56 | 7.96 | 5.08 |
|  | [22] | 194 | 6.31 | 6.60 | 7.63 |
|  | [24] | 14 | 5.15 | 6.31 | 7.24 |
|  | [25] | 8 | 5.33 | 7.27 | 6.58 |
|  | [26] | 80 | 5.50 | 5.90 | 7.61 |
|  | [27] | 36 | 7.51 | 8.09 | 7.55 |
|  | [29] | 100 | 6.95 | 7.46 | 7.57 |
|  | [30] | 42 | 7.02 | 7.98 | 7.36 |
|  | [31] | 51 | 6.68 | 7.12 | 7.60 |
|  | [32] | 200 | 4.68 | 4.93 | 7.64 |
|  | [33] | 30 | 7.79 | 8.75 | 7.37 |
|  | [34] | 17 | 7.30 | 9.10 | 6.70 |
|  | [35] | 20 | 10.80 | 12.71 | 6.60 |
| CT inlet | [5] | 41 | 9.59 | 10.81 | 3.54 |
|  | [21] | 240 | 8.75 | 9.05 | 4.15 |
|  | [22] | 194 | 8.13 | 8.51 | 4.12 |
|  | [23] | 120 | 8.20 | 8.80 | 4.01 |
|  | [26] | 80 | 6.35 | 6.75 | 4.11 |
|  | [27] | 36 | 10.67 | 11.33 | 3.98 |
|  | [36] | 40 | 8.68 | 9.76 | 3.66 |
|  | [37] | 150 | 8.10 | 8.76 | 3.98 |
|  | [39] | 106 | 7.96 | 8.64 | 3.96 |
|  | [40] | 30 | 7.44 | 8.37 | 3.79 |
|  | [41] | 56 | 8.03 | 8.97 | 3.78 |
|  | [42] | 150 | 8.10 | 8.76 | 3.98 |
|  | [43] | 40 | 8.93 | 9.67 | 3.92 |
|  | [44] | 21 | 7.49 | 8.62 | 3.62 |
|  | [45] | 50 | 8.76 | 9.90 | 3.60 |
|  | [46] | 59 | 8.24 | 9.36 | 3.63 |
|  | [47] | 42 | 9.50 | 10.71 | 3.53 |
|  | [48] | 38 | 10.48 | 12.52 | 2.77 |
|  | [49] | 41 | 7.92 | 8.04 | 4.19 |
|  | [50] | 60 | 8.84 | 10.46 | 3.16 |
|  | [51] | 18 | 9.02 | 12.58 | 1.63 |
|  | [52] | 40 | 7.16 | 8.72 | 3.22 |
|  | [53] | 50 | 9.08 | 10.32 | 3.51 |
|  | [54] | 40 | 7.13 | 7.87 | 3.92 |
|  | [55] | 43 | 6.70 | 7.30 | 4.02 |
|  | [56] | 55 | 8.34 | 8.86 | 4.05 |
|  | [58] | 23 | 8.07 | 8.23 | 4.17 |
| CT outlet | [5] | 41 | 9.42 | 10.58 | 10.08 |
|  | [21] | 240 | 8.65 | 8.97 | 10.45 |
|  | [26] | 80 | 6.25 | 6.65 | 10.44 |
|  | [27] | 36 | 9.27 | 9.93 | 10.35 |
|  | [36] | 40 | 8.31 | 9.35 | 10.16 |
|  | [38] | 40 | 11.10 | 12.90 | 9.56 |
|  | [50] | 60 | 8.37 | 10.10 | 9.62 |
|  | [52] | 40 | 7.69 | 9.55 | 9.49 |
|  | [54] | 40 | 7.50 | 8.50 | 10.18 |
|  | [57] | 17 | 8.01 | 9.69 | 9.67 |
